# Multifocal transcranial electrical stimulation to enhance cognitive functions: a systematic review

**DOI:** 10.3389/fnins.2025.1698215

**Published:** 2025-12-17

**Authors:** Ji-Soo Choi, Won Kee Chang, Won-Seok Kim, Nam-Jong Paik

**Affiliations:** Department of Rehabilitation Medicine, Seoul National University, Seoul National University Bundang Hospital, Seongnam-si, Republic of Korea

**Keywords:** brain stimulation, neuromodulation, multifocal, transcranial electric stimulation, transcranial current stimulation, cognition

## Abstract

**Background:**

Multifocal transcranial Electrical Stimulation (tES) has emerged as an innovative approach for modulating cognitive functions by concurrently targeting multiple brain regions. Despite its potential, the efficacy and underlying mechanisms of this technique remain unclear.

**Objective:**

To evaluate the efficacy of multifocal tES in enhancing cognitive function by examining targeted brain regions, stimulation protocols, and behavioral and neurophysiological outcomes.

**Methods:**

A systematic search of PubMed, EMBASE, Cochrane Library, Scopus, and Web of Science databases was conducted up to February 10, 2025, following the Preferred Reporting Items for Systematic Reviews and Meta-Analyses 2020 guidelines. The inclusion criteria encompassed human studies utilizing concurrent dual-site or multifocal tES with pre- and post-intervention behavioral assessments. Studies on deep brain stimulation, corticocortical paired associative stimulation, sequential stimulation, case reports, and reviews were excluded.

**Results:**

Of the 1,453 initial records, 14 met the inclusion criteria. The studies predominantly employed transcranial direct current stimulation/transcranial alternating current stimulation, targeting the frontoparietal network. Neurophysiological data from electroencephalography and functional magnetic resonance imaging revealed network-level modulations. Behavioral outcomes were inconsistent, with some studies reporting improvements in executive function, working memory, and response inhibition, whereas others showed no significant advantages over sham stimulation.

**Conclusion:**

Although multifocal tES is a promising modality, clearly determining its efficacy is currently limited by heterogeneous study designs, small sample sizes, and conflicting findings. Future research should prioritize multi-arm trials, incorporate neurophysiological biomarkers, and develop personalized stimulation protocols to optimize the effectiveness of this technique.

**Trial registration:**

PROSPERO Registration # CRD420250646196.

## 1 Introduction

Transcranial Electrical Stimulation (tES) has garnered considerable attention for its potential applications in neuroscience and clinical settings, emerging as a promising tool to enhance functional recovery in individuals with neurological disorders (Schulz et al., 2013; Bhattacharya et al., 2022). tES, including transcranial direct current stimulation (tDCS) and transcranial alternating current stimulation (tACS) has been extensively applied for neurological disorders, such as stroke (Yan et al., 2020; Hara et al., 2021; Li et al., 2024), neurodegenerative diseases (Pini et al., 2018; Sanches et al., 2020), mood disorders, and schizophrenia (Sabé et al., 2024). Although promising results have been reported in various domains of cognitive functions (Yan et al., 2020; Begemann et al., 2020; Hara et al., 2021), the clinical and behavioral outcomes remain heterogeneous, posing challenges in establishing consistent effects and identifying optimal candidates (Sanches et al., 2020).

Several factors contribute to these challenges, including a limited understanding of the precise mechanisms underlying tES, variations in the participants’ brain functional states, and insufficient knowledge of the neural activity responsible for specific functions (Boes et al., 2018; Bhattacharya et al., 2022). There is growing recognition that complex cognitive functions are not confined to a single brain region but rather depend on the simultaneous activation and synchronization of multiple brain networks (Sale et al., 2015). Multifocal tES has been proposed as a novel approach that targets functional networks rather than isolated brain regions (Fischer et al., 2017). Unlike traditional monofocal tES, which primarily modulates activity in a focal brain region, multifocal tES aims to enhance network-level interactions, potentially leading to more robust functional improvements (Menardi et al., 2022). Multifocal stimulation protocols can modulate network-level dynamics by selectively inhibiting or facilitating activity across two or more regions—for example, by alternating inhibitory and excitatory stimulation using tDCS at different sites, or in the case of tACS, by adjusting the relative phase between electrodes to probe and manipulate inter-regional oscillatory coupling. However, despite its conceptual advantages, the current evidence base for multifocal tES remains limited. Only a small number of studies have applied multifocal protocols in clinical populations, and most available findings originate from experiments conducted in healthy adults focusing on behavioral outcomes.

Despite growing interest in multifocal tES, systematic reviews of its clinical and behavioral outcomes are lacking. Therefore, this review aimed to synthesize the existing literature on multifocal tES concerning cognitive functions, evaluate the current level of evidence, and explore future research directions. Specifically, we sought to identify (1) the brain regions targeted, (2) the stimulation methods employed (e.g., stimulation parameters, electrode montages), and (3) the observed outcomes.

We excluded studies involving high-definition stimulation targeting a single region, as they fall outside the scope of this review. Corticocortical paired associative stimulation studies were excluded, as they involve sequential stimulation with a fixed interstimulus interval to induce Hebbian plasticity, which has been comprehensively reviewed elsewhere (Di Luzio et al., 2024; Hernandez-Pavon et al., 2023). We excluded sequential stimulation paradigms that prime one brain region before another, along with studies applying simple bilateral homologous areas stimulation.

## 2 Materials and methods

### 2.1 Eligibility criteria

This systematic review was conducted in accordance with the PRISMA 2020 guidelines (Page et al., 2021). It includes peer-reviewed research articles and preprints published in English that investigated the effects of multifocal NIBS involving human participants. Eligible studies were required to report behavioral outcomes related to cognitive functions including domains of memory, attention, executive function, working memory, language, visuospatial processing and emotion assessed pre- and post-intervention (Sachdev et al., 2014). Simulation studies, case reports, retrospective studies, review articles, and non-research articles were excluded.

### 2.2 Information sources, search strategy, and selection process

A comprehensive literature search was conducted across five principal databases (PubMed, EMBASE, Cochrane Library, Scopus, and Web of Science) covering all available records from database inception to the final search date on February 10, 2025. No restrictions on publication year were applied. Detailed search terms are provided in Supplementary material 1. The selection of studies was performed by two independent reviewers. Titles and abstracts were screened to exclude irrelevant studies, followed by full-text assessment for eligibility.

#### 2.2.1 Data collection process

Data extraction was independently conducted by two reviewers utilizing a standardized data extraction form. Discrepancies were resolved through discussion until consensus was achieved.

#### 2.2.2 Risk of bias assessment

The methodological quality of the included studies was assessed using the Cochrane Risk of Bias (RoB) tool (Higgins et al., 2011). Each domain was rated as having low, high, or unclear RoB, based on the information provided in the articles. Two independent reviewers conducted the assessments, and disagreements were resolved through consultation with a third reviewer.

## 3 Results

**Figure 1:**
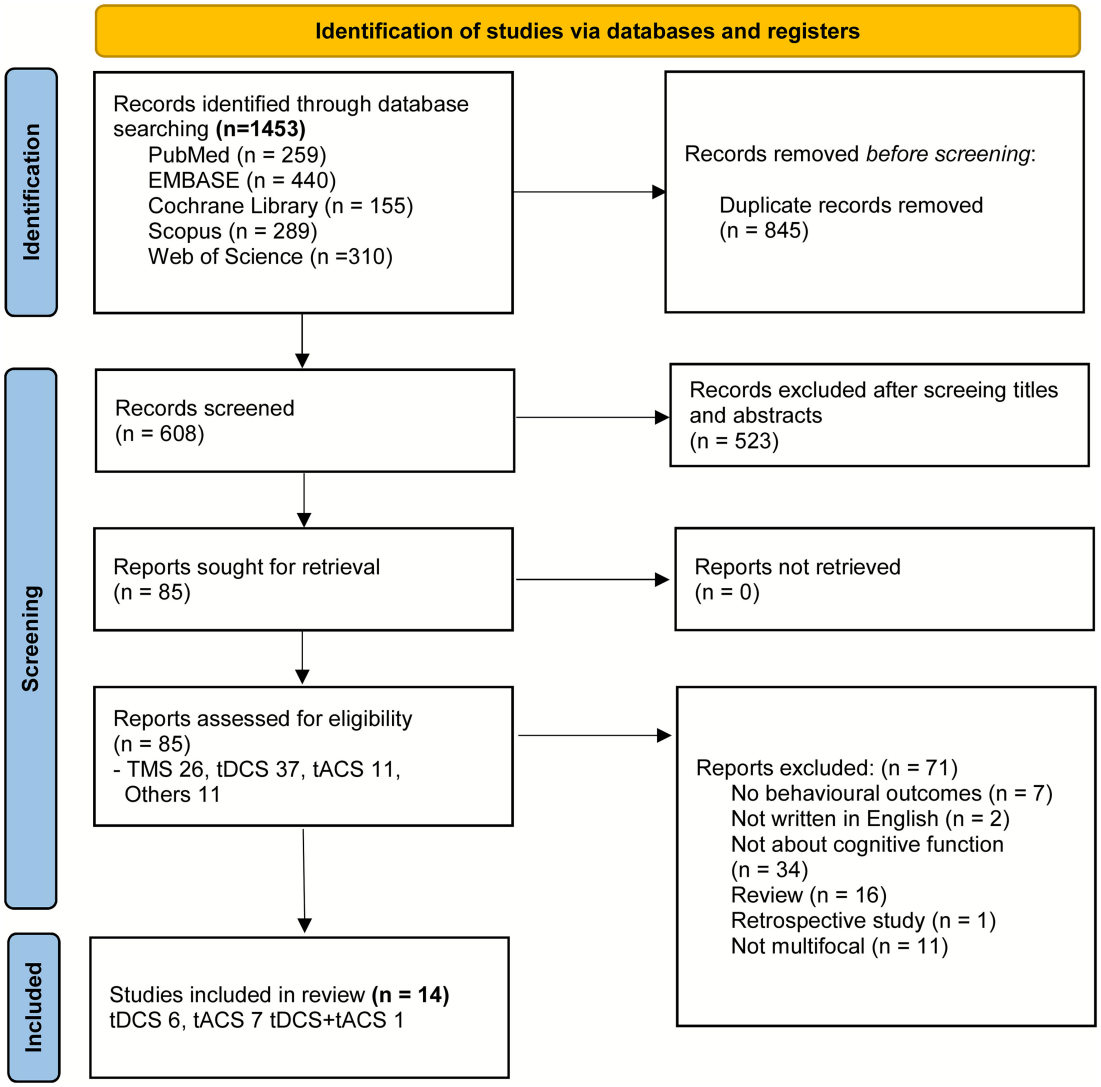
PRISMA flow chart diagram for study selection process.

The study selection process is illustrated in Figure 1. A total of 1,453 records were identified through the database search. After the removal of duplicates, 608 records remained and were screened by their titles and abstracts. In total, 85 full-text articles were assessed for eligibility. Of these, 71 were excluded, resulting in 14 studies utilizing tDCS (*n* = 6), tACS (*n* = 7), and a comparison of tDCS and tACS (*n* = 1) being included in the review.

**Figure 2:**
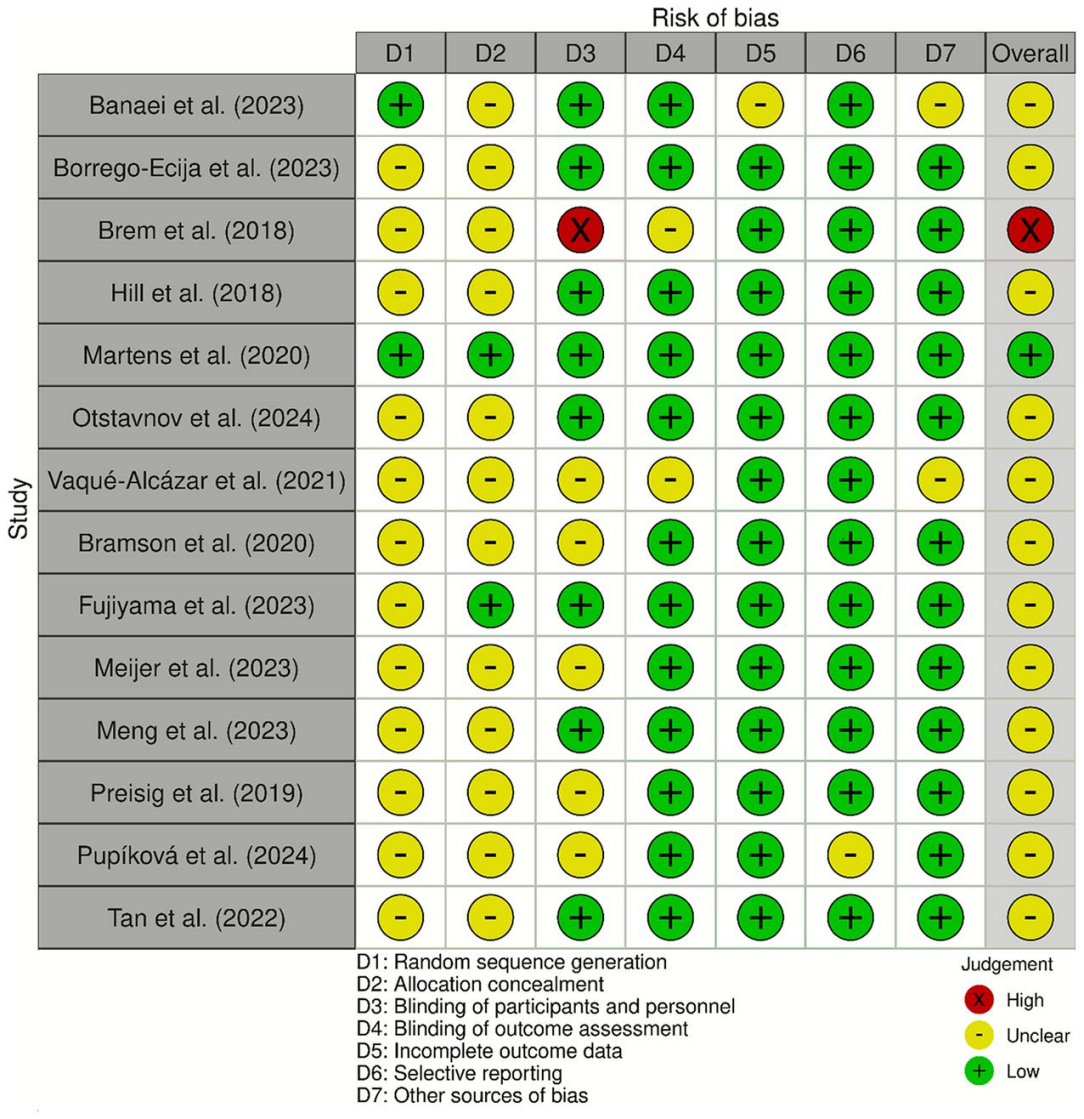
Risk of bias assessment of the included studies.

The RoB varied across the included studies (Figure 2). Most studies exhibited a low RoB in domains related to random sequence generation and incomplete outcome data. However, allocation concealment and blinding of participants were frequently rated as unclear or high-risk due to insufficient methodological details. Selective reporting was judged as unclear in many cases, due to the unavailability of pre-registered protocols.

### 3.1 Multifocal tDCS targeting cognitive function

Seven studies that investigated the effects of multifocal tDCS on cognitive functions are summarized in Tables 1 and 2 (Brem et al., 2018; Hill et al., 2018; Martens et al., 2020; Vaqué-Alcázar et al., 2021; Banaei et al., 2023; Borrego-Écija et al., 2023; Otstavnov et al., 2024). Most studies targeted the frontoparietal network.

**Table 1:** Multifocal transcranial direct current stimulation (tDCS) studies targeting cognitive function.

Study	Intervention	Stimulation montage	Intensity	Duration	Target function	Participants	Design	Session
Banaei et al. (2023)	Offline tDCS (with chocolate)	l-M1: C3-AF4; l-DLPFC: F3-(between Fpz and AFz)	2 mA	20 min	Visual choice reaction; endurance	10 male cyclists (age 23.5 ± 1.13)	Crossover DC + tDCS DC + sham WC + tDCS WC + sham	1 (1 wk. washout)
Borrego-Écija et al. (2023)	Online tDCS	Anode: (F7, FC1, FC5, P7) Cathode: (C1, Fpz, PO8)	< 2 mA (per electrode)	26 min	Language	15 PPA patients (10F, age 63.6 ± 8.7)	Crossover ST + tDCS ST + sham	10/2 wks (3 mo washout)
Brem et al. (2018)	Online tDCS, tACS	mftDCS anode: (F3, F4, P3, P4) cathode: (Fz, T7, P8, Oz) mftACS: in-phase: (F3, F4, P3, P4) anti-phase: (Fz, PO7, PO8)	<1 mA (per electrode)	30 min	Fluid intelligence	82 healthy adults (36Fs, age 29.46 ± 12.09)	Parallel mftACS: 17 mftDCS: 15 tDCS (F3-AF8): 17 tRNS (F3-F4): 16 control: 17	9 / 9 days
Hill et al. (2018)	Offline tDCS	DLPFC: F3–(Fp1, Fz, C3, F7) Parietal: P3—(P7, Pz)	1.5 mA	15 min	Working memory	16 healthy adults (10F, age 32.81 ± 10.80)	Crossover dual-site DLPFC sham	1 (≥2 days washout)
Martens et al. (2020)	Offline tDCS	Anode: (F3, F4, CP5, CP6) Cathode: (Fp2, FPz, O1, Oz)	1 mA (per electrode)	20 min	Awareness	46 chronic DOC patients (19F, age 46.02 ± 14.91)	Crossover active -sham	1 (≥2 days washout)
Otstavnov et al. (2024)	Offline tDCS	l-DLPFC: F3– (F1, AF3, F5, FC3) l-PPC: P3—(P1, CP3, P5, PO3)	1 mA (per active electrode)	15 min	Working memory	47 healthy young adults (24F, age 25.2 ± 2.8)	Parallel dual-site: 16 DLPFC: 16 sham: 15	1
Vaqué-Alcázar et al. (2021)	Online tDCS	Compensatory anode: (AF7, F4, FC5, P3, P4) cathode: (P7, P8, Cz) Maintenance anode: (AF3, C3, C4, F4) cathode: (FC6, Fpz, Oz, Cz)	< 2 mA (per electrode)	25 min	Working memory	24 elderly 12 stable, 12 declined cognition (14F, age 71.62 ± 2.65)	Crossover compensatory maintenance sham	1 (1 mo washout)

DC, dark chocolate; DLPFC, dorsolateral prefrontal cortex; DOC, disorder of consciousness; F, females; M1, primary motor cortex; mftACS, multifocal transcranial alternating current stimulation; mftDCS, multifocal tDCS; PPA, primary progressive aphasia; PPC, posterior parietal cortex; ST, speech therapy; tRNS, transcranial random noise stimulation; WC, white chocolate.

**Table 2:** Results of multifocal transcranial direct current stimulation (tDCS) studies targeting cognitive function.

Study	Main outcome measurement	Measurement timing	Behavioural result	Neurophysiological result
Banaei et al. (2023)	Choice reaction time (CRT) task during and after Time-to-exhaustion (TTE) task; surface EMG	POST (immediate)	TTE↓ by ‘DC + tDCS’ vs. WC + tDCS (d = 0.77) and WC + sham (d = 0.85); During and Post-TTE CRT↓ by ‘DC + tDCS’ (vs. WC + sham) (d = 2.4)	VM EMG amplitude↑ by ‘DC + tDCS’ and ‘DC + sham’ (vs. ‘WC + sham’) (ηρ^2^ = 0.344)
Borrego-Écija et al. (2023)	Language battery; resting fMRI (BOLD)	POST (immediate); POST (1 month); POST (3 months)	No inter-group difference	Rt frontal medial and bilateral paracingulate BOLD ↑ by active tDCS (vs sham)
Brem et al. (2018)	Combined Gf score (BOMAT, RAPM, Sandia matrices)	POST (immediate)	Gf ↑ by the mftDCS, tDCS, tRNS (vs. control)	N/A
Hill et al. (2018)	N-back, DST, Corsi block tapping; TEP (N40, P60, N100, P200 at Cz); N-back task-related EEG (power)	POST (5 min); POST (30 min)	No inter-group difference (ηρ^2^ = 0.003)	Post-30 min P60↑ by dual-site and mono-site tDCS (vs. sham)
Martens et al. (2020)	CRS-R; resting EEG (power, LZW complexity)	POST (immediate)	No inter-group difference	θ and δ complexity ↑ by active tDCS (vs. sham) ΔCRS-R ∝ post-tDCS *θ* and *δ* complexity (negatively) ΔCRS-R ∝ baseline θ complexity in MCSandEMCS (negatively)
Otstavnov et al. (2024)	OSPAN (MA, CA, CT, RT)	POST (5 min, total <45 min)	MA↑(ηρ^2^ = 0.032), CT↓(ηρ^2^ = 0.009) by dual-site tDCS (vs. DLPFC, vs. sham tDCS) RT↓ by dual-site and DLPFC tDCS (vs. sham) (ηρ^2^ = 0.045)	N/A
Vaqué-Alcázar et al. (2021)	N-back task (d’, RT); task-related fMRI (BOLD)	During intervention	No inter-group difference	Occipital BOLD ↓by compensatory tDCS in decliner group (vs. sham)

BOMAT, Bochumer–Matrizen test; CA, calculation accuracy; CRS-R, coma recovery scale-revised; CT, calculation time; d’, accuracy; DC, dark chocolate; DST, digit span test; EEG, electroencephalography; EMG, electromyography; Gf, fluid intelligence; LZW, Lempel–Ziv –Welch; MA, memory accuracy; mftDCS, multifocal tDCS; OSPAN, operation span task; POST, post-intervention; RAPM, Raven’s Advanced Progressive Matrices; RT, response time; TEP, transcranial magnetic stimulation-evoked potential; tRNS, transcranial random noise stimulation; VM, vastus medialis; WC, white chocolate.

Three studies (Hill et al., 2018; Vaqué-Alcázar et al., 2021; Otstavnov et al., 2024) investigated working memory, employing varying protocols. Hill et al. (2018) focused on the dorsolateral prefrontal cortex (DLPFC) and parietal area using a high-definition tDCS montage in healthy adults. The N-back test with concurrent electroencephalography (EEG) and transcranial magnetic stimulation (TMS)-evoked potentials was performed pre-, 5 min post-, and 30 min post-stimulation. Digit span and Corsi block tapping tasks were performed pre- and 30 min post-stimulation. The effects of the 15-min dual-site stimulation were compared to those of DLPFC mono-site and sham stimulations. Conversely, Vaqué-Alcázar et al. (2021) categorized older participants into two groups (“stable” and “decliner”) based on changes in the N-back test performance over 4 years. Based on a previous study that identified two functional magnetic resonance imaging (fMRI) patterns in cognitively intact older adults (Fernández-Cabello et al., 2016). Two multi-electrode montages, targeting frontoparietal networks were developed. During the 25-min stimulation period, N-back tasks and fMRI were conducted for approximately 11 min. Both studies assessed the effects of a single session per condition, with neither demonstrating significant differences in working memory performance compared to sham. In terms of neurophysiological outcomes, Hill et al. (2018) reported significantly increased P60 amplitudes in TMS-evoked potentials 30 min after dual-site and DLPFC-only stimulation compared to that of sham, indicating alterations in cortical excitability. Only dual-site stimulation resulted in an increase in the N100 compared to baseline, although this was not significantly different from sham. During the 2-back task, dual-site stimulation increased frontocentral theta power and parietal gamma power 5 min post-stimulation; however, these effects were not statistically significant compared to the sham condition. Vaqué-Alcázar et al. (2021) observed that in a group with declining cognition, compensatory montage reduced activity in the right lingual gyrus, bilateral occipital, and lateral-occipital cortex, regions that exhibited elevated baseline activity compared to the stable group.

Contrary to these null behavioral findings, Otstavnov et al. (2024) reported significant improvements in working memory performance following dual-site stimulation. Utilizing a parallel-group design, they compared the effects of simultaneous anodal high-definition tDCS over DLPFC and posterior parietal cortex to DLPFC-only and sham conditions in 48 healthy adults. Working memory was evaluated using the OSPAN task, which integrates short-term memory retention with arithmetic processing. The entire task was completed within 45 min of stimulation and remained within the expected offline effect window. A significant effect of stimulation type was observed for memory accuracy and calculation time. Post-hoc comparisons revealed that dual-site stimulation significantly reduced calculation time compared to mono-site (−0.88 ± 0.19 s, *p* = 0.0001) and sham (−1.01 ± 0.19 s, *p* = 0.0001) stimulations. Memory accuracy was also significantly higher in the dual-site group than in the mono-site (+5.99% ± 1.78%, *p* = 0.003) and sham (+4.57% ± 1.81%) groups. Both active stimulation groups exhibited reduced recall response times compared to the sham group, although no significant difference was observed between the dual-site and mono-site.

Banaei et al. (2023) evaluated 10 male cyclists under conditions of hypoxia and mental fatigue, employing dual-site anodal tDCS targeting the DLPFC and primary motor cortex (M1) or sham for 20 min. Participants also consumed either dark chocolate (DC) or white chocolate (WC) preceding each session. A group comparison was made between the type of chocolate and stimulation. Post-stimulation, endurance was measured using a time-to-exhaustion (TTE) task, and the visual choice reaction time (CRT) was measured during and after the TTE task. The “DC + tDCS” group exhibited significantly enhanced endurance compared to the “WC + tDCS” (Cohen’s d = 0.77, *p* = 0.002) and “WC + sham” groups (d = 0.85, *p* = 0.038) and displayed faster visual CRT during (d = 2.4, *p* = 0.008) and post-TTE (d = 1.04, *p* = 0.016) compared to the “WC + sham” group. However, no significant differences in CRT or TTE were identified among “WC + tDCS,” “WC + sham,” or “DC + sham” groups, suggesting that neither dual-site tDCS nor DC supplementation alone accounted for the observed effects. Additionally, no neurophysiological variables demonstrated any significant effect of tDCS.

Brem et al. (2018), the sole parallel design study, evaluated fluid intelligence using a five-arm comparison: multifocal tDCS (mftDCS), multifocal tACS (mftACS), tDCS, transcranial random noise stimulation (tRNS), and a control group. The montages for mftDCS and mftACS targeted the bilateral frontoparietal network using eight electrodes, with mftACS set at 40 Hz. Each session lasted 30 min, with concurrent gamified executive function training administered daily over nine sessions. Significant improvements in fluid intelligence were observed in the mftDCS, tDCS, and tRNS groups compared to the control group, but not in the mftACS group. Compared to mftACS, mftDCS (*p* = 0.043) and tDCS (*p* = 0.029) showed significant improvements, whereas tRNS achieved a marginal effect (*p* = 0.067). Logistic regression revealed similar beta coefficients for mftACS (0.179 ± 0.088) and tDCS (0.182 ± 0.083).

Two studies (Martens et al., 2020; Borrego-Écija et al., 2023) focused on the patient population. Borrego-Écija et al. (2023) investigated 15 individuals with primary progressive aphasia using a montage of seven electrodes targeting the language network in the left hemisphere. The intervention comprised 10 sessions over 2 weeks, and in each session, speech therapy with online stimulation was conducted for 26 min. Language assessments were conducted, but no significant differences between active and sham tDCS were observed at any assessment time point. Resting-state fMRI revealed increased activity in the right frontal medial cortex and bilateral paracingulate gyri in the active tDCS condition compared to the sham group.

Martens et al. (2020) examined 46 individuals with chronic disorders of consciousness (DOC). An 8-electrode montage was used to activate the bilateral frontoparietal network, with 20 min of stimulation per session. Awareness was assessed using the Coma Recovery Scale-Revised (CRS-R), and resting EEG data were analyzed for band power and complexity using the Lempel–Ziv–Welch (LZW) algorithm. Clinical improvements were observed in nine participants, but no significant differences were identified between active and sham stimulations. Among the 42 participants with EEG data, tDCS had no effect on EEG relative band power but significantly increased LZW complexity in the theta and delta bands. While these changes did not translate into behavioral outcomes, post-tDCS theta and delta complexities showed a negative correlation with CRS-R changes, and baseline theta complexity was negatively correlated with CRS-R changes in the Conscious group.

### 3.2 Dual-site tACS targeting cognitive function

Seven studies targeting cognitive function investigated the effects of tACS (Preisig et al., 2019; Bramson et al., 2020; Meijer et al., 2023; Tan et al., 2025; Meng et al., 2023; Fujiyama et al., 2023; Pupíková et al., 2024), each employing dual-site stimulation within a single session (Tables 3 and 4). Of these, five studies (Preisig et al., 2019; Bramson et al., 2020; Meijer et al., 2023; Meng et al., 2023; Pupíková et al., 2024) administered online tACS during cognitive task performance, whereas one study (Tan et al., 2025) measured the effects of offline tACS post-stimulation, and the other (Fujiyama et al., 2023) compared the outcomes of both methodologies. The sinusoidal nature of tACS necessitates careful selection of the stimulation site and the frequency band associated with the targeted function. Additionally, phase dependence between two stimulation sites can result in varying effects (Vosskuhl et al., 2018; Fehér et al., 2022). One study exclusively employed in-phase tACS, wherein stimulation at both sites was synchronized in the same phase (Fujiyama et al., 2023), while the remaining studies compared the effects of in-phase tACS with those of anti-phase tACS in which stimulation at both sites was delivered with a phase difference of 180°.

**Table 3:** Dual-site transcranial alternating current stimulation (tACS) studies targeting cognitive function.

Study	Intervention	Stimulation montage	Intensity	Frequency	Duration	Target function	Participants	Design	Session
Bramson et al. (2020)	Online tACS	r-aPFC: MNI [26, 54, 0]; l-SMC: MNI [−28, −32, 64] (center-surround)	2 mA	6 Hz (θ) at aPFC; 75 Hz (γ) at SMC (AM in 6 Hz envelope)	1 min × 8 blocks	Emotion-action control	41 healthy male (age 23.8 ± 3.4)	Crossover in-phase anti-phase sham	1 (≥1 day washout)
Fujiyama et al. (2023)	In-phase tACS	r-IFG: INT of Fz-T4 and Cz-F8; preSMA: Fz (2 × 1 electrodes)	1 mA	20 Hz (β)	15 min	Response inhibition	53 healthy adults (32F, age 23.07 ± 5.07)	Parallel online: 18 offline: 18 sham: 17	1
Meijer et al. (2023)	Online tACS	r-aPFC: MNI [26, 54, 0]; l-SMC: MNI [−28, −32, 64] (center-surround)	2 mA	6 Hz (θ) at aPFC; 75 Hz (γ) at SMC (AM in 6 Hz envelope)	1 min × 8 blocks	Emotion-action control	49 adults with social anxiety (age 24.0 ± 4.3)	Crossover in-phase anti-phase sham	1 (1wk washout)
Meng et al. (2023)	Online tACS	r-IFG: FC6—FP1; r-M1: C4—FP1	2 mA	20 Hz (β)	20 min	Response inhibition	Exp1: 69 healthy adults (21F, age 21.59 ± 1.44) Exp2: 25 healthy adults (12F, age 21.48 ± 1.00)	Exp1: Parallel in-phase: 25 anti-phase: 24 sham: 20 Exp2: Parallel in-phase: 15 sham: 10	1
Preisig et al. (2019)	Online tACS	l-IFG: between FT7 and FC5; l-STG: between P7 and P5 (center-surround)	1 mA	4 Hz (θ)	Not specified	Language (Auditory-motor mapping)	19 healthy adults (13F, mean age 22 ± 2.33)	Crossover in-phase anti-phase sham	2 (pooled) (no washout)
Pupíková et al. (2024)	Onlie tACS	r-MFG: MNI [36, 28, 36] r-IPL: MNI [38, −50, 36] (center-surround) ; individualized from task-fMRI	3 mA (Exp 1)	Exp 1: 4.17–4.98 Hz (θ) Exp 2: 4.32–5.28 Hz (θ), 30.06–37.04 Hz (γ) ; individualized from task-EEG	20 min	Working memory	Exp 1: 20 healthy adults (9F, age 64.49 ± 6.98) Exp 2: 20 healthy adults (12F, age 67.6 ± 4.70)	Exp 1: Crossover (θ frontoparietal tACS) frontal only parietal only in-phase anti-phase sham Exp 2: Crossover (frontoparietal tACS) θPre-γPar γPre-θPar θPre-γBurst γBurst-θPar sham	1 (1 day washout)
Tan et al. (2025)	Offline tACS	r-IFG: INT of Fz-T4 and Cz-F8; preSMA: Fz (2 × 1 electrodes)	1 mA	20 Hz (β)	20 min	Response inhibition	18 healthy young (11F, age 23.56 ± 4.49); 15 healthy elderly (10F, age 68.80 ± 5.39)	Crossover in-phase anti-phase	1 (1wk washout)

AM, amplitude modulated; aPFC, anterior prefrontal cortex; DCM, dynamic causal modeling; EC, effective connectivity; EEG, electroencephalography; ER, error rate; F, females; FC, functional connectivity; IFG, inferior frontal gyrus; ImCoh, imaginary component of coherency; INT, intersection; IPL, inferior parietal lobule; M1, primary motor cortex; MFG, middle frontal gyrus; preSMA, pre-supplementary motor area; SMC, sensorimotor cortex; SSRT, stop-signal reaction time; STG, superior temporal gyrus; WPLI, weighted phase lag index.

**Table 4:** Results of dual-site transcranial alternating current stimulation (tACS) studies targeting cognitive function.

Study	Main outcome measurement	Measurement timing	Behavioral result	Neurophysiological result
Bramson et al. (2020)	Social approach-avoidance task (congruency effect on ER); task-related fMRI (BOLD, aPFC→SMC EC by DCM)	During intervention	ΔER↓ by in-phase tACS (ηρ^2^ = 0.13)	aPFC BOLD↓ ∝ ΔER↓ by in-phase tACS (ηρ^2^ = 0.19) EC↓ ∝ ΔER↓ by in-phase tACS
Fujiyama et al. (2023)	Stop-signal Task (SSRT); resting and task-related EEG (preSMA-rIFG FC by ImCoh)	POST online: immediate offline: >20 min	SSRT↓ by offline tACS (vs. online tACS) (d = 1.51)	Resting FC in β↑ ∝ SSRT↓ by online tACS (d = 0.10) Resting FC in *α*↓, β↑ by online tACS Task-related FC in β↑ by online tACS (vs offline, sham) Task-related FC in α↑, β↓, γ↓ by offline tACS;
Meijer et al. (2023)	Social approach-avoidance task (congruency effect on ER); task-related fMRI (BOLD)	During intervention	Not specified	DLPFC BOLD↑ ∝ ΔER↓ by in-phase tACS
Meng et al. (2023)	Stop-signal task (SSRT); resting EEG (FC by WPLI in inhibitory control network)	During intervention; POST (immediate)	SSRT↓ after stimulation by in-phase tACS (vs. anti-phase (ηρ^2^ = 0.13)and sham tACS. (ηρ^2^ = 0.13))	C4-PO4 FC in β↑ (vs. sham) by in-phase tACS ∝ SSRT↓ (ηρ^2^ = 0.14)
Pupíková et al. (2024)	N-back task (d’, RT); resting fMRI (FC, Exp 1 only)	N-back task: During intervention; fMRI: POST (immediate)	Exp 1. (vs. sham) d’↑ in 3-back task by frontal tACS. (d = 0.30) RT↓ in 3-back task by frontal (d = 0.27), parietal (d = 0.286), in-phase fronto-parietal (d = 0.40) tACS Exp 2. (vs. sham) d’↓ in 2-back task by γBurst-θPar tACS (d = 0.49). d’↓ in 3-back task by γBurst-θPar, θPre-γBurst, γPre-θPar tACS. (d = 0.464) RT↓ in 2-back task by γPre-θPar, γBurst-θPar tACS (d = 0.40) RT↓ in 3-back task by θPre-γPa, γBurst-θPar tACS. (d = 0.27)	Exp 1. aPFC-DLPFC FC↓ by frontal tACS (vs sham) (not correlated to online behavioural change)
Preisig et al. (2019)	Verbal repetition performance (under 6 phase lags for the 4 Hz auditory stimuli)	During intervention	No significant effect	N/A
Tan et al. (2025)	Stop-signal task (SSRT); resting and task-related EEG (rIFG-preSMA FC by ImcoH)	POST (immediate)	SSRT↓ by in-phase and anti-phase tACS	No significant effect

AM, amplitude modulated; aPFC, anterior prefrontal cortex; d’, accuracy; DCM, dynamic causal modeling; EC, effective connectivity; EEG, electroencephalography; ER, error rate; F, females; FC, functional connectivity; IFG, inferior frontal gyrus; ImCoh, imaginary component of coherency; INT, intersection; M1, primary motor cortex; POST, post-intervention; preSMA, pre-supplementary motor area; RT, response time; SMC, sensorimotor cortex; SSRT, stop-signal reaction time; STG, superior temporal gyrus; WPLI, weighted phase lag index.

Three studies examined the effects of 20 Hz tACS on response inhibition, as measured by the stop-signal task (SST) under in-phase and anti-phase conditions (Tan et al., 2025; Meng et al., 2023; Fujiyama et al., 2023). Fujiyama et al. (2023) and Tan et al. (2025) targeted the right inferior frontal gyrus (IFG) and the pre-supplementary motor area (preSMA) using identical montages (Tan et al., 2025; Meng et al., 2023; Fujiyama et al., 2023). Tan et al. (2025) investigated the effects of offline tACS without sham conditions by comparing in-phase and anti-phase tACS. Fujiyama et al. (2023) used a parallel design to compare online, offline, and sham tACS in a larger cohort using in-phase tACS. Meng et al. (2023) targeted the right IFG and M1 with slightly different electrode placement using a current intensity of 2 mA. None of the three studies reported significant effects on reaction time for the go signal during the SST. However, significant improvements in the stop-signal reaction time (SSRT) were observed, although the specific effects varied. In Tan et al. (2022), in-phase and anti-phase tACS conditions exhibited significant SSRT reductions compared to baseline (significant main effect of time, *p* = 0.008; no significant interaction effects), with no observable EEG changes. In Fujiyama et al. (2023), the offline group exhibited significantly faster SSRT than the online group (d = −1.51, *p* = 0.009), but not compared to the sham group (d = −0.48, *p* = 0.958). Notably, online tACS increased resting-state functional connectivity in the beta band (d = 0.10, *p* < 0.001), which significantly correlated with SSRT improvement (r = −0.49, *p* = 0.038). Meng et al. (2023) demonstrated phase dependency, with significant SSRT improvement observed exclusively in the in-phase condition compared to both sham (ηp^2^ = 0.13, *p* = 0.01) and anti-phase (ηp^2^ = 0.13, *p* = 0.01) conditions. Additionally, in-phase tACS significantly increased functional connectivity in the C4-PO4 region of the inhibitory control network compared to sham (ηp^2^ = 0.14, *p* = 0.01), with the magnitude of this increase correlating with SSRT improvements (r = −0.46, *p* = 0.03).

Two studies employed online tACS targeting the frontal pole and sensorimotor cortex using the same montage, leveraging theta-gamma phase coupling to examine emotion-action control through a social approach-avoidance task (Bramson et al., 2020; Meijer et al., 2023). A theta-band sinusoidal current (6 Hz) was applied to the prefrontal cortex, while a gamma-band current (75 Hz) with a 6 Hz amplitude-modulated envelope was applied to the sensorimotor cortex. The relationship between the phase of the prefrontal tACS and that of the sensorimotor tACS envelope was manipulated across three conditions: in-phase, anti-phase, and sham, using a crossover design. The task assessed the ability to suppress the tendency to choose positive facial expressions over negative ones and to adhere to instructions, particularly in incongruent conditions where emotional tendencies conflicted with the instructions. The objective was to determine whether tACS could mitigate the congruency effect (higher error rates in incongruent conditions) by targeting the networks and oscillatory frequencies identified in previous research. To address inter-participant variability in tACS effects, the studies avoided simple group-level comparisons and used blood oxygen level-dependent (BOLD) signal changes within the region of interest as a proxy for the effective tACS dose. Behavioral outcomes were subsequently analyzed to identify dose-dependent relationships within each group. Bramson et al. (2020) conducted the study among healthy male students. Only in-phase tACS suppressed the congruency effect in a dose-dependent manner, linked to reduced prefrontal activity and decreased connectivity to the sensorimotor cortex. In a follow-up study, Meijer et al. (2023) examined participants with high social anxiety, a group known for impaired emotion-action control. Instead of focusing on electrode placement sites, the study measured activity in the DLPFC, which is known to be reduced in individuals with high social anxiety. The results revealed that in-phase tACS increased BOLD activity in the DLPFC and higher BOLD values were associated with improved emotion-action control, demonstrating a dose–response relationship.

Pupíková et al. (2024) conducted two crossover experiments to evaluate the impact of frontoparietal tACS on white matter in healthy older adults. In the first experiment, which included five theta-tACS conditions, frontal monofocal stimulation improved the 3-back accuracy (d = 0.30, *p* = 0.004), while response speed increased in all active conditions except the anti-phase (frontal: d = 0.27, *p* = 0.0183; parietal: d = 0.286, *p* = 0.0107; in-phase: d = 0.40, *p* = 0.0001). These effects were observed solely under conditions of high cognitive load. Resting-state fMRI revealed reduced connectivity between the right anterior and dorsolateral prefrontal cortices following frontal stimulation, although this was not correlated with behavioral outcomes.

In the second experiment, the five-theta and gamma cross-frequency stimulation conditions were examined. The only condition that enhanced response speed in the 3-back task without compromising accuracy was the prefrontal theta combined with continuous parietal gamma (d = 0.24, *p* = 0.02). All other conditions resulted in a speed–accuracy trade-off. Prefrontal gamma bursts with parietal theta produced the most consistent trade-off, improving speed (2-back: d = 0.40, *p* = 0.0001; 3-back: d = 0.27, *p* = 0.02), but reducing accuracy (2-back: d = 0.49, *p* < 0.0001; 3-back: d = 0.4640, *p* < 0.0001). Prefrontal continuous gamma with parietal theta improved 2-back speed (d = 0.42, *p* < 0.0001), but impaired 3-back accuracy (d = 0.2787, *p* = 0.0132). Lastly, the prefrontal theta with parietal gamma bursts only led to reduced 3-back accuracy (d = 0.5748, *p* < 0.0001), without any speed benefit. These findings underscore the specificity of the dual-site tACS effects, contingent on spatial configuration, frequency pairing, and task demands.

Preisig et al. (2019) explored the effects of high-definition tACS on auditory-motor mapping by targeting the left IFG and superior temporal gyrus. Utilizing a crossover design, this study compared the verbal repetition performance across in-phase, anti-phase, and sham tACS conditions. Participants were presented with three-syllable non-word stimuli at a rate of 4 Hz, while online tACS was administered at the same frequency with six different time lags relative to the auditory stimuli. Repetition performance was analyzed using a two-way repeated measures analysis of variance to evaluate the effects of the stimulation and phase lag conditions. The results revealed no significant main effects of the stimulation condition or interaction effects between stimulation condition and phase.

## 4 Discussion

### 4.1 Multifocal tDCS targeting cognitive function

Research on tDCS aimed at enhancing cognitive function has predominantly focused on the frontoparietal network (Brem et al., 2018; Hill et al., 2018; Martens et al., 2020; Vaqué-Alcázar et al., 2021; Otstavnov et al., 2024). However, the behavioral outcomes have generally been negligible. Although two studies reported significant effects of indeterminate origins (Brem et al., 2018; Banaei et al., 2023), a recent investigation employing a dual-site frontoparietal montage alongside a cognitively demanding task (Otstavnov et al., 2024) demonstrated robust improvements in working memory performance directly attributable to multifocal stimulation. Conversely, neurophysiological measurements using fMRI or EEG frequently reveal stimulation-modulated activity in regions associated with the targeted function. Borrego-Écija et al. (2023) identified a distant effect, wherein stimulation of the left hemisphere led to increased activation of the right frontal medial cortex and other areas within the language-processing network. Similarly, Vaqué-Alcázar et al. (2021) observed that in a group with cognitive decline over 4 years, activity in the occipital area—which had been compensatively elevated compared to a stable group—was suppressed by tDCS using a “compensatory montage.” This montage was designed to replicate the fMRI patterns observed in older adults with preserved N-back task performance based on previous research (Fernández-Cabello et al., 2016).

Otstavnov et al. (2024) provide an informative contrast. By employing a more demanding executive-loaded working memory task (OSPAN), they were able to detect behavioral improvements following dual-site stimulation, which were not observed in studies using N-back paradigms. Their findings suggested a functional dissociation within the frontoparietal network, with frontal stimulation selectively enhancing retrieval speed and parietal involvement supporting information storage and processing. Although neurophysiological measurements were not included, the observed behavioral pattern aligns with the findings of Hill et al. (2018), wherein dual-site stimulation increased frontoparietal theta and gamma power. This suggests that behavioral effects may emerge more clearly when stimulation is paired with tasks that robustly engage distributed executive control networks.

The inability of neurophysiological changes to translate into behavioral outcomes can be interpreted in various ways. The limitations may include low dosage, short intervention period, or the ceiling effect in healthy groups. In lesioned brains, such as in individuals with stroke or DOC, interhemispheric inhibition may interfere with stimulation effects (Boddington and Reynolds, 2017) or baseline heterogeneity may result in inappropriate participant selection. Specifically, Martens et al. (2020) demonstrated that tDCS influences EEG complexity, and that baseline complexity is associated with behavioral outcomes. These findings highlight the importance of biomarkers to identify suitable target groups. Furthermore, Hill et al. (2018) observed electrophysiological changes 30 min post-stimulation, indicating that delayed effects might be overlooked if measurements are restricted to the immediate post-intervention period. Lastly, small sample sizes across studies may have reduced the statistical power, further limiting the detection of significant effects.

### 4.2 Dual-site tACS targeting cognitive function

Most tACS studies targeting cognitive function aimed at improving inhibitory executive function by stimulating the prefrontal motor areas yielded significant outcomes (Bramson et al., 2020; Meijer et al., 2023; Tan et al., 2022; Meng et al., 2023; Fujiyama et al., 2023). These studies employed dual-site stimulation with single sessions per condition, without comparison to single-site stimulation. In tACS, phase dependency is a central focus, and significant effects are predominantly observed in the in-phase condition (Bramson et al., 2020; Meijer et al., 2023; Meng et al., 2023; Fujiyama et al., 2023). In-phase tACS is hypothesized to synchronize the neural activity between two targeted regions, thereby enhancing the overall function of the associated network (Fehér et al., 2022), which likely contributes to the observed improvements in behavioral outcomes.

The mechanisms underlying the neuromodulatory effects of tACS vary depending on the stimulation paradigm employed (Vosskuhl et al., 2018). Online tACS possibly acts via entrainment and phase coupling, whereas the delayed effects of offline tACS are attributable to entrainment and plasticity-related changes (Vogeti et al., 2022). Among the four studies applying online tACS, three reported significant results (Bramson et al., 2020; Meijer et al., 2023; Meng et al., 2023). Fujiyama et al. (2023) found offline tACS to be more effective than online tACS in enhancing behavioral outcomes. However, EEG revealed that changes in resting-state functional connectivity were significantly correlated with behavioral improvements only in the online tACS condition. Although both types of tACS were administered at beta frequency, task-related connectivity in the beta band was modulated in opposite directions: online tACS enhanced connectivity, while offline tACS reduced it. This suggests distinct neurophysiological effects based on whether tACS was delivered online or offline.

In studies targeting the SSRT by stimulating the right IFG and preSMA or M1, beta-band frequencies were utilized (Meng et al., 2023; Fujiyama et al., 2023). Conversely, studies targeting emotion-action control by stimulating the right anterior PFC and left SMA utilized theta-gamma coupling (Bramson et al., 2020; Meijer et al., 2023). These frequency selections were based on prior studies identifying the oscillatory activity associated with the target function and its underlying network (Picazio et al., 2014; Bramson et al., 2018). The observed increases in functional connectivity within the stimulated frequency bands have been significantly associated with improvements in behavioral outcomes in some studies (Meijer et al., 2023; Fujiyama et al., 2023), highlighting the potential importance of frequency selection in achieving effective neuromodulation. In contrast, the negative results reported by Preisig et al. (2019) may be partly attributable to a mismatch between the targeted function and chosen frequency band, suggesting that the studied function may be associated with a different frequency range. These findings highlight the critical role of frequency tuning in tACS in optimizing its neuromodulatory effects.

Two studies examining the congruency effect (Bramson et al., 2020; Meijer et al., 2023) explored phase coupling across different frequencies and assessed outcomes using fMRI. Despite employing identical montages, their studies yielded divergent results, contingent upon the studied population. In a study of healthy participants (Bramson et al., 2020), inhibition of anterior prefrontal activity enhanced control abilities. In contrast, in a study involving an anxiety group characterized by diminished DLPFC activity (Meijer et al., 2023), an increase in DLPFC activity was associated with improved control abilities. These findings indicate that the neurophysiological effects of neuromodulation on the network vary depending upon the disease state, underscoring the importance of considering population-specific neural dynamics.

In contrast to studies focusing on inhibitory control, Pupíková et al. (2024) expanded dual-site tACS research into the domain of working memory, demonstrating that frontoparietal theta stimulation enhanced response speed, with accuracy benefits emerging solely with prefrontal theta. However, these accuracy gains were disrupted when theta was paired with parietal gamma bursts, whereas continuous gamma preserved the effect, highlighting that temporal structure and interregional coupling influenced behavioral outcomes. Notably, reduced frontoparietal connectivity was observed in resting-state fMRI, but did not correlate with performance, suggesting that neural modulation might not directly translate into behavioral change. These results emphasize the need for precision in multifocal tACS design regarding phase alignment and stimulation mode.

Findings across the included multifocal tES studies suggest that cognitive functions can be conceptualized as network-level phenomena, with effective modulation occurring along frontotemporal and frontoparietal pathways that involve the dorsolateral prefrontal cortex, posterior parietal cortex, and bilateral M1. Nevertheless, neurophysiological outcomes varied substantially across studies and individuals. One likely contributor to this variability is the heterogeneity in anatomical and physiological characteristics—such as cortical thickness, gyrification patterns, white-matter integrity, and endogenous oscillatory frequencies (Wischnewski et al., 2023; Hunold et al., 2023). These inter-individual differences strongly influence the distribution of electric fields and the extent of achievable network modulation. Incorporating personalized elements—such as individualized head modeling, structural or functional connectivity mapping, or tailoring tACS frequencies to intrinsic oscillatory peaks—may therefore enhance biomarker–behavior coupling and increase the precision of tES interventions.

Another notable observation is that multifocal tES studies predominantly employed high-definition tES (HD-tES), with only one tDCS study and one tACS study using conventional montages (Banaei et al., 2023; Meng et al., 2023). This pattern emerged although our search strategy did not restrict stimulation protocols to HD-tES. The predominance of HD-tES likely reflects its methodological advantages for multifocal stimulation. In particular, multi-channel HD-tES configurations provide substantially greater focality and enable more precise current steering compared with conventional electrode montages, especially when implementing tDCS (Masina et al., 2021). Nonetheless, direct comparative studies examining HD versus conventional montages across both multifocal tDCS and tACS are still limited, and further investigations are needed to determine whether the preference for HD-tES represents true neurophysiological superiority or simply reflects current methodological conventions in the field.

In line with the preceding discussion, our findings can be interpreted within the narrower context of methodological considerations in multifocal tES. Prior studies have reported that tES often produces variable cognitive effects, in part due to its relatively diffuse current distribution and limited temporal specificity (Parkin et al., 2015; Thut et al., 2017). Although these characteristics may reduce its consistency compared with more focal techniques such as TMS (Polanía et al., 2012), emerging evidence indicates that HD-tES can enhance focality and current steering through multi-channel electrode configurations (Edwards et al., 2013). These methodological developments underscore the importance of optimized montage design and individualized current-flow modeling as potential strategies for reducing variability in multifocal tES outcomes. Nevertheless, further large-scale studies involving patient populations are required to confirm the efficacy of these approaches.

Many studies included in our review demonstrated an unclear or high risk of bias in blinding, consistent with prior reports noting insufficient description of blinding procedures in early tES research (Antal et al., 2017; Bikson et al., 2018). This concern is particularly relevant for crossover designs, where differences in sham credibility may influence participants’ expectations and thereby affect behavioral outcomes (Turner et al., 2021). Because sensations associated with active stimulation can sometimes compromise blinding, rigorous and well-reported sham protocols—as well as explicit assessment of sham credibility—are essential for minimizing bias in future studies.

In conclusion, studies on multifocal tES targeting cognitive functions have primarily focused on prefrontal-motor area combinations and frontoparietal networks. Among the various techniques, tACS leveraging phase dependency has demonstrated particularly promising effects in modulating inhibitory executive function. However, many studies possess methodological limitations, unclear isolation of multifocal stimulation, and lack supporting neurophysiological evidence. To address these limitations, future studies should implement multi-arm designs and incorporate neurophysiological measurements to enhance the interpretation of results. Establishing protocols that effectively link neural network activity and connectivity changes to behavioral outcomes is crucial for advancing the field. Moreover, because the present analysis was restricted to studies using tES, the conclusions regarding multifocal stimulation necessarily remain confined to this modality. A broader framework will require integrating evidence from other NIBS techniques that offer superior spatial or temporal precision. For example, several meta-analyses suggest that rTMS can yield consistent cognitive benefits, particularly in memory-related domains, with medium effect sizes reported across both healthy and clinical populations (Xie et al., 2021; Chou et al., 2020; Liao et al., 2015). ccPAS has been shown to modulate specific cortico-cortical connections, and recent systematic reviews and meta-analyses (Di Luzio et al., 2024; Hernandez-Pavon et al., 2023) suggest more robust and predictable cognitive-connectivity effects than standard tES. In addition, emerging technologies such as temporal interference stimulation (TIS) have recently been reviewed, showing promise for non-invasive targeting of deeper brain regions via intersecting high-frequency electric fields, although at present most evidence comes from modelling and animal studies and translation to human cognition remains preliminary (Guo et al., 2023). A recent study by Missey et al., have reported multifocal TIS to bilateral hippocampus and temporal lobe can modulate the working memory according to the stimulation frequency and location (Missey et al., 2025). Likewise, transcranial ultrasound stimulation (TUS) demonstrates the capacity to modulate sub-cortical structures with fine spatial precision (Sarica et al., 2022). Because each modality engages distinct neurophysiological mechanisms—from synaptic coincidence and plasticity in ccPAS, to envelope-wave steering in TIS, to mechano-electrical modulation in LIFU—future comparative studies of multifocal tES alongside these techniques may reveal divergent outcome patterns. Integrating such evidence will be essential for positioning multifocal stimulation within the full NIBS landscape and for determining whether outcome variability reflects intrinsic modality limits or is largely attributable to methodological optimization.
