# Supplementary material for: Multifocal transcranial electrical stimulation to enhance cognitive functions: a systematic review
Source: Front Neurosci. 2025 Dec 17;19:1698215. doi: 10.3389/fnins.2025.1698215 (PMC12753878; doi:10.3389/fnins.2025.1698215)
Supplement: Supplementary file 1 [file Data_Sheet_1.DOCX]

***Supplementary Material***

**1 Supplementary Data: Search strategy**

**Pubmed**

((("non-invasive"[Title/Abstract] OR "noninvasive"[Title/Abstract]) AND "stimulation"[Title/Abstract]) OR (("transcranial"[Title/Abstract] OR "trans-cranial"[Title/Abstract]) AND "stimulation"[Title/Abstract]) OR ("tDCS"[Title/Abstract] OR "tACS"[Title/Abstract] OR "TMS"[Title/Abstract] OR "rTMS"[Title/Abstract] OR "transcranial direct current stimulation"[MeSH Terms] OR "transcranial magnetic stimulation"[MeSH Terms]) OR ("theta-burst"[Title/Abstract] OR "theta-burst"[Title/Abstract] OR "TBS"[Title/Abstract] OR "cTBS"[Title/Abstract] OR "iTBS"[Title/Abstract]) OR ("temporal interference stimulation"[Title/Abstract] OR "TIS"[Title/Abstract] OR "tTIS"[Title/Abstract]) OR ("focused-ultrasound"[Title/Abstract] OR "focused-ultrasound"[Title/Abstract] OR "LIFU"[Title/Abstract])) AND ("dual-site"[Title/Abstract] OR "dual-site"[Title/Abstract] OR "multifocal"[Title/Abstract] OR "multi-focal"[Title/Abstract])

**Cochrane**

((("non-invasive" OR "noninvasive") AND "stimulation") OR (("transcranial" OR "trans-cranial") AND "stimulation") OR (("tDCS" OR "tACS" OR "TMS" OR "rTMS")) OR (("theta burst" OR "theta-burst" OR "TBS" OR "cTBS" OR "iTBS")) OR (("temporal interference stimulation" OR "TIS" OR "tTIS")) OR (("focused ultrasound" OR "focused-ultrasound" OR "LIFU"))) AND (("dual-site" OR "dual site" OR "multifocal" OR "multi-focal")):ti,ab,kw

**EMBASE**

(((“non-invasive”:ti,ab,kw OR “noninvasive”:ti,ab,kw) AND “stimulation”:ti,ab,kw) OR ((“transcranial”:ti,ab,kw OR “trans-cranial”:ti,ab,kw) AND “stimulation”:ti,ab,kw) OR (“tdcs”:ti,ab,kw OR “tacs”:ti,ab,kw OR “tms”:ti,ab,kw OR “rtms”:ti,ab,kw) OR (“theta burst”:ti,ab,kw OR “theta-burst”:ti,ab,kw OR “tbs”:ti,ab,kw OR “ctbs”:ti,ab,kw OR “itbs”:ti,ab,kw) OR (“temporal interference stimulation”:ti,ab,kw OR “tis”:ti,ab,kw OR “ttis”:ti,ab,kw) OR (“focused ultrasound”:ti,ab,kw OR “focused-ultrasound”:ti,ab,kw OR “lifu”:ti,ab,kw)) AND (“dual-site”:ti,ab,kw OR “dual site”:ti,ab,kw OR “multifocal”:ti,ab,kw OR “multi-focal”:ti,ab,kw)

**Scopus**

(TITLE-ABS-KEY ((((("non-invasive" OR "noninvasive") AND "stimulation") OR (("transcranial" OR "trans-cranial") AND "stimulation") OR (("tDCS" OR "tACS" OR "TMS" OR "rTMS")) OR (("theta burst" OR "theta-burst" OR "TBS" OR "cTBS" OR "iTBS")) OR (("temporal interference stimulation" OR "TIS" OR "tTIS")) OR (("focused ultrasound" OR "focused-ultrasound" OR "LIFU"))) AND ("dual-site" OR "dual site" OR "multifocal" OR "multi-focal"))))

**Web of Science**

TS=((((("non-invasive" OR "noninvasive") AND "stimulation")OR(("transcranial" OR "trans-cranial") AND "stimulation")OR(("tDCS" OR "tACS" OR "TMS" OR "rTMS"))OR(("theta burst" OR "theta-burst" OR "TBS" OR "cTBS" OR "iTBS"))OR(("temporal interference stimulation" OR "TIS" OR "tTIS"))OR(("focused ultrasound" OR "focused-ultrasound" OR "LIFU")))AND("dual-site" OR "dual site" OR "multifocal" OR "multi-focal")))
